# Current landscape of hospital information systems in gynecology and obstetrics in Germany: a survey of the commission Digital Medicine of the German Society for Gynecology and Obstetrics

**DOI:** 10.1007/s00404-023-07223-1

**Published:** 2023-09-23

**Authors:** André Pfob, Sebastian Griewing, Katharina Seitz, Christoph Hillen, Sven Becker, Christian Bayer, Uwe Wagner, Peter Fasching, Markus Wallwiener, For the Kommission Digitale Medizin, Deutsche Gesellschaft für Gynäkologie und Gebursthilfe (DGGG), Harald Abele, Matthias Alexa, Jan Philipp Cieslik, Dominik Dannehl, Thomas Deutsch, Tanja Fehm, Oliver Graupner, Max Hackelöer, Andreas Hartkopf, Alexander Hein, Maike Henninsen, Martin Hirsch, Sascha Hoffmann, Hanna Hübner, Elsa Hollatz-Galuschki, Bernadette Jäger, Heike Janse, Fran Kainer, Maria M. Karsten, Marion Kiechle, Claus Richard Lattrich, Andreas Schmutzler, Elke Schulmeyer, Eric Steiner, Lea Louise Volmer, Stephanie Wallwiener, Jan Weichert, Martin Weiß, Armin Wöckel

**Affiliations:** 1 Department of Obstetrics & Gynecology, Heidelberg University Hospital, Im Neuenheimer Feld 440, 69120 Heidelberg, Germany; 2 National Center for Tumor Diseases (NCT), German Cancer Research Center (DKFZ), Heidelberg, Germany; 3 Department of Gynecology and Obstetrics, University Hospital Marburg, Philipps-University Marburg, Baldingerstraße, 35043 Marburg, Germany; 4 Comprehensive Cancer Center Erlangen-EMN, Department of Gynecology and Obstetrics, University Hospital Erlangen, Friedrich-Alexander University Erlangen-Nuremberg, Erlangen, Germany; 5 Department of Gynecology and Gynecologic Oncology, University Medical Center Hamburg-Eppendorf, Hamburg, Germany; 6 Department of Gynecology, University Hospital, Frankfurt am Main, Germany; 7 WMC HEALTHCARE GmbH, Munich, Germany

**Keywords:** eHealth, Digital health, Artificial intelligence, Implementation, Gynecology and obstetrics

## Abstract

**Purpose:**

Hospital information systems (HIS) play a critical role in modern healthcare by facilitating the management and delivery of patient care services. We aimed to evaluate the current landscape of HIS in the specialty of gynecology and obstetrics in Germany.

**Methods:**

An anonymous questionnaire was distributed via the German Society of Gynecology and Obstetrics newsletter in December 2022. The questionnaire covered the domains baseline demographic information, satisfaction with daily use, satisfaction with implementation, and degree of digitization.

**Results:**

Ninety-one participants completed the survey. Median age was 34 years; 67.4% (60 of 89) were female, and 32.6% (29 of 89) were male. Of the survey participants, 47.7% (42 of 88) were residents, 26.1% (23 of 91) senior physicians, and 9.1% (8 of 88) medical directors. The degree of digitization of clinical documentation is mainly mixed digital and paper-based (64.0%, 57 of 89) while 16.9% (15 of 89) operate mainly paper-based. The current HIS has been in use on average for 9 years. The median number of different software systems used in daily routine is 4. About 33.7% (30 of 89) would likely or very likely recommend their current HIS to a colleague.

**Conclusions:**

The current landscape of HIS in gynecology and obstetrics in Germany is characterized by a high heterogeneity of systems with low interoperability and long service life; thus, many healthcare professionals are not satisfied. There is both a need to enhance and an interest in modernizing the technological infrastructure to meet today’s requirements for patient care.

## What does this study add to the clinical work?

**Table:** 

The current landscape of hospital information systems in gynecology and obstetrics in Germany is characterized by a high heterogeneity of systems with low interoperability and long service life. Thus, many healthcare professionals are not satisfied with these systems.	

## Background

Hospital information systems (HIS) play a critical role in modern healthcare by facilitating the management and delivery of patient care services. These systems integrate various components, including electronic health records (EHRs), computerized physician order entry (CPOE), clinical decision support systems (CDSS), and administrative and financial modules, to streamline healthcare processes and enhance the quality of care. In Germany, significant attention has been directed to implementing and utilizing HIS in recent years, with the aim of improving patient outcomes, increasing efficiency, and ensuring better coordination among healthcare providers.

Germany, renowned for its robust healthcare system, is currently struggling to keep up with the speed of digitalization in healthcare [1]. The German healthcare system is characterized by a mix of statutory and private health insurance schemes, with a focus on providing universal coverage and high-quality care to its population. However, with a strong emphasis on data protection, security, and privacy, implementing HIS in Germany requires compliance with strict legal and regulatory requirements, such as the Federal Data Protection Act (Bundesdatenschutzgesetz) and the European General Data Protection Regulation (GDPR). Despite the numerous advantages of modern HIS, implementing and utilizing the systems present several challenges here. These include issues related to data privacy and security, standardization of data formats, integration with existing healthcare systems, and the need for user training and acceptance [2]. Additionally, the financial investments required for implementing and maintaining HIS represent a significant hurdle for healthcare organization [3].

By considering digital healthcare in general, we neglect the fact that each speciality has distinct needs. Indeed, little is known about the current landscape of HIS in the medical field of gynecology and obstetrics in Germany. By launching the “Commission for Digital Medicine”, the German Association of Gynecology and Obstetrics (DGGG) is confronting these developments and aims to translate them into action by accelerating the digitization of gynecological and obstetric care. Therefore, the interuniversity working group set a starting point by conducting a qualitative survey to evaluate the current landscape of HIS in gynecology and obstetrics in Germany. The objective of the questionnaire was to identify the HIS used in inpatient gynecological and obstetric care, and to assess satisfaction with current data storage standards.

## Materials and methods

### Participant recruitment and selection

Participants were recruited by distributing the anonymous questionnaire via the German Society of Gynecology and Obstetrics (DGGG, Deutsche Gesellschaft für Gynäkologie und Geburtshilfe) newsletter in December 2022 (English translation of the questionnaire in supplementary material). The research was conducted in accordance with the precepts established by the Helsinki Declaration.

### Survey questionnaire

The survey questionnaire is composed of questions determined by an expert panel within the commission with respect to the use of HIS in gynecology and obstetrics in Germany. The domains covered are aligned with baseline demographic information, satisfaction with daily use, satisfaction with implementation, and degree of digitization. The final questionnaire was approved by common consent during an in-person meeting of the commission Digital Medicine of the German Society for Gynecology and Obstetrics on July 1, 2022.

### Statistical analysis

Survey responses were subjected to descriptive statistical assessment, using absolute values and relative frequencies. Analyses were performed using R software (R Foundation for Statistical Computing, Version 4.1).

### Role of the funding source

There was no funding source for this analysis and the resulting report.

## Results

### Demographics of survey participants

In all, 91 participants completed the survey. In terms of demographic distribution, the median age was 34 years; 67.4% (60 of 89) were female, and 32.6% (29 of 89) were male. With respect to their current working positions, 47.7% (42 of 88) were residents, 26.1% (23 of 91) senior physicians, and 9.1% (8 of 88) medical directors. Further details are displayed in Table 1.

**Table 1:** Baseline characteristics of survey participants

Characteristic	Value
Age—years	
Median (25, 75 percentile)	34.0 (30.0, 46.25)
Gender—no. (%)	
Female	60(67.4)
Male	29(32.6)
No answer	2
Working position—no. (%)	
Resident (Assistenz*ärztin)	42 (47.7)
Attending (Fach*ärztin)	10 (11.4)
Senior physician (Ober*ärztin)	23 (26.1)
Medical director (Chef*ärztin)	8 (9.1)
Physician in private practice (Niedergelassener Fach*ärztin)	3 (3.3)
Honorarium physician (Honorar*ärztin)	2 (2.2)
No answer	3
Worksite—no. (%)	
University hospital	38 (43.2)
Maximum care hospital	8 (9.1)
Medium care hospital	14 (15.9)
Basic care hospital	16 (18.2)
Specialized hospital	4 (4.5)
Private practice	8 (9.1)
Missing	3

### Current HIS

**Fig. 1:**
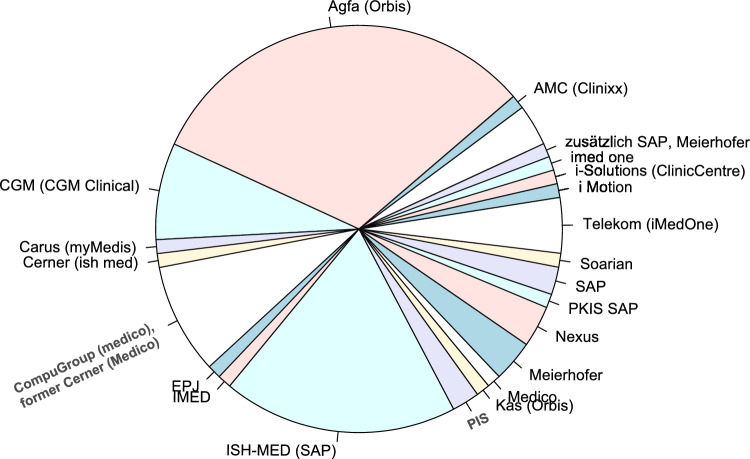
Current hospital information systems in obstetrics and gynecology in Germany

Figure 1 illustrates the distribution of HIS currently in use in the specialty of gynecology and obstetrics in Germany. The five most commonly used systems are Orbis (Agfa) 32.6% (29 of 89), ISH-med (SAP) 19.1% (17 of 89), Medico (Compu Group) 9% (8 of 89), CGM clinical (CGM) 7.9% (7 of 89), and iMedOne (Telekom) 4.5% (4 of 89).

Table 2 summarizes further details regarding the current HIS: The degree of digitization of clinical documentation is mainly mixed digital and paper-based (64.0%, 57 of 89) while 16.9% (15 of 89) operate mainly paper-based systems. On average, the respective HIS has been in use for 9 years (25th percentile 4; 75th percentile 15). The median number of different software systems used in daily routine amounts to 4 (25th percentile 3; 75th percentile 5). The degree of automated data transfer between systems is mainly mixed automated and manual transfer (53.6%, 37 of 69) while 40.6% (28 of 69) mainly transfer data manually.

**Table 2:** Current status of hospital information systems in Germany in obstetrics and gynecology

Characteristic	Value
Degree of digitalization of clinical documentation—no. (%)	
Fully digitalized	17 (19.1)
Mixed digital and paper-based	57 (64.0)
Paper based	15 (16.9)
No answer	2
Number of years hospital has used the current hospital information system––Median (25th, 75th percentile)	9 (4, 15)
Number of different software systems used in daily routine––Median (25th, 75th percentile)	4 (3, 5)
Degree of automated data transfer between systems—no. (%)	
Fully automated data transfer	4 (5.8)
Mixed automated and manual transfer	37 (53.6)
Manual data transfer	28 (40.6)
No answer	21
Use of speech recognition systems for documentation—no. (%)	
Yes	23 (33.3)
No	46 (66.7)
No answer	3 21

### Satisfaction with current HIS

**Fig. 2:**
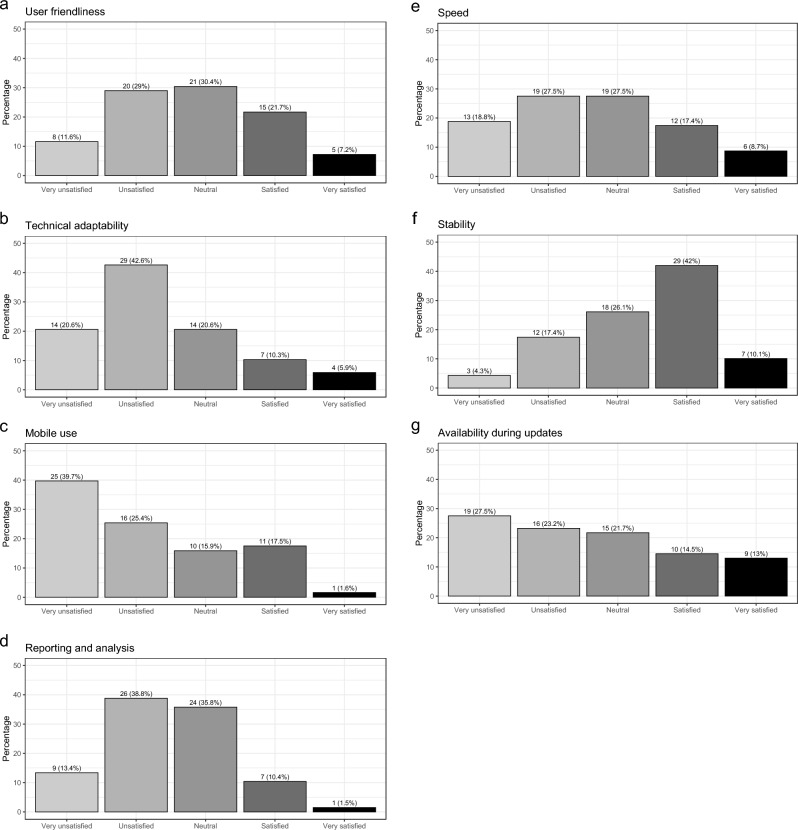
Satisfaction during daily routine use of hospital information system

**Fig. 3:**
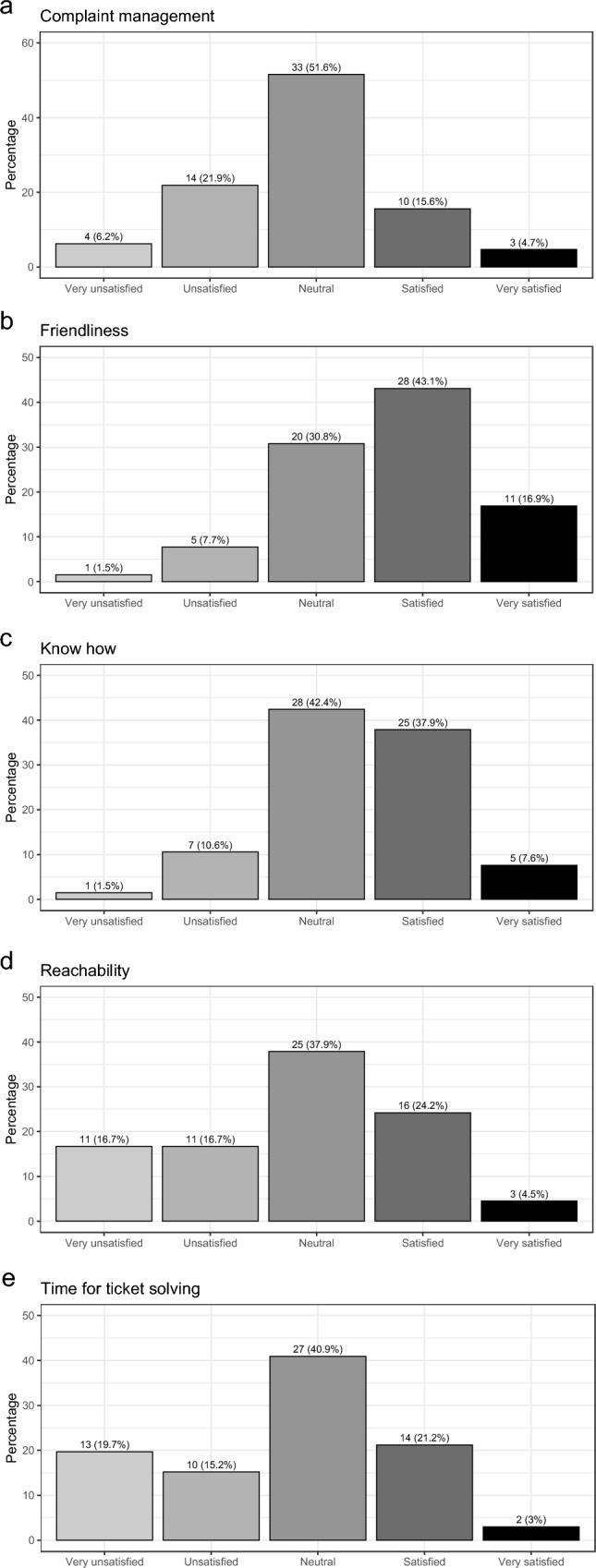
Satisfaction with user support of hospital information system

About 33.7% (30 of 89) would likely or very likely recommend their current HIS to a colleague. Figure 2 summarizes user satisfaction regarding daily routine use of the software; satisfaction with the user support is summarized in Fig. 3.

With respect to routine use of the software, the majority is satisfied or very satisfied with the stability (52.1%) whereas the majority is unsatisfied or very unsatisfied with the software availability during updates (50.7%), technical adaptability (62.6%), reporting and analytical capability (52.2%), and mobile use (65.1%). A neutral attitude was observed for functionally, speed, and user friendliness.

With respect to user support for the software, the majority is satisfied or very satisfied with the friendliness of the support team (60.0%). A neutral attitude was observed for complaint management, knowhow of the support team, reachability, and time for ticket solving.

Among the survey participants, six medical directors provided further details regarding satisfaction with implementation of the HIS at their hospital. Details are summarized in Table 3. The top three reasons provided for implementing a new system were: centralized documentation of medical data (83.3%, 5 of 6), legal requirements (66.7%, 4 of 6), and increase in effectiveness (50%, 3 of 6).

**Table 3:** Satisfaction with implementation of hospital information system by medical directors

How satisfied are you with the following aspects of the hospital information system implementation	Very unsatisfied	Unsatisfied	Neutral	Satisfied	Very satisfied
Speed	1 (16.7)	3 (50.0)	1 (16.7)	0 (0.0)	1 (16.7)
Size of implementation team	0 (0.0)	3 (50.0)	2 (33.3)	1 (16.7)	0 (0.0)
Knowhow of implementation team	0 (0.0)	3 (50.0)	1 (16.7)	1 (16.7)	1 (16.7)
Project management	1 (20.0)	0 (0.0)	2 (40.0)	2 (40.0)	0 (0.0)
Introduction to software	1 (16.7)	3 (50.0)	1 (16.7)	1 (16.7)	0 (0.0)
Availability of implementation team	1 (16.7)	3 (50.0)	1 (16.7)	0 (0.0)	1 (16.7)

## Discussion

In this study, we applied a qualitative questionnaire to evaluate the current state of HIS use in the specialty of gynecology and obstetrics in Germany. The data indicate a pronounced heterogeneity in HIS use in this specific medical field. Furthermore, the identified median time of 9 years for HIS use indicates that the pace of technological progress and change is particularly slow. Heterogeneity not only prevails in terms of HIS, but also with regard to subsystems. As such, the median number of different software systems used by gynecological and obstetric practitioners in Germany in daily routine is 4. The finding that about 41% use a manual data transfer between these subsystems confirms that there is an interface problem with lacking interoperability. Still, about 17% operate mainly paper-based in their clinical setting while only 34% would recommend their HIS to a colleague or friend. Nevertheless, more than a third of the respondents identify as digital innovators and the vast majority acknowledge the potential of digital medicine to drive efficiency in daily routine care.

These findings create a feeling of dissonance between the desired, yet possible, and the actual state of HIS use in gynecology and obstetrics in Germany. Since technological innovations continue to transform the world, sometimes even ushering in a whole new era and with entirely new standards for the future, many areas of our daily life have changed and become faster, more efficient, or simply easier [4]. With regard to modern HIS, several benefits for both healthcare providers and patients are expected: Efficient electronic documentation and storage of patient records reduce reliance on paper-based records, minimizing the risk of errors and enabling quick access to up-to-date information. Electronic prescribing and medication management systems enhance medication safety, reduce medication errors, and support decision-making by providing alerts and reminders to healthcare professionals. Clinical decision support systems integrated within HIS assist healthcare providers in making evidence-based decisions, improving the accuracy and appropriateness of diagnoses and treatments. Furthermore, HIS enables data-driven quality improvement initiatives and supports population health management by providing comprehensive data for analysis and monitoring. The digital assessment shows that this rich array of benefits is recognized and acknowledged by practitioners in the field as 78.4% (69 of 88) agreed or strongly agreed that digital medicine can help to reduce the increasing workload. Nevertheless, while technological advancement shapes entire industries, the clocks in healthcare appear to tick more slowly [5]. This has proven to be particularly true in Germany, where digital innovation in healthcare is often held back by high levels of regulation. According to a 2018 international study, Germany ranked 16th out of 17 for technical implementation and use of medical data. It was shown that there are regional offerings with great potential for innovation in our federally organized structures, but that nationwide digitization is in its infancy compared to our neighbors [1]. These data confirm that this general impression holds true for the specialty of gynecology and obstetrics. Indeed, the HIS of German practitioners in this specialty are 9 years old on average, require a considerable degree of manual data transfer and documentation, and only one third of survey participants are satisfied with their HIS, confirming that the pace of technological progress is particularly slow and calls for change.

Nevertheless, the year 2023 continues to show the status quo of a paralyzed German healthcare system in need of efficiency improvement [6]. German healthcare has been shown to be the second most expensive system in an OECD comparison, accounting for 12.8% of GDP spent on health expenditure [7]. Its low efficiency and consequentially high costs are primarily derived from problems revolving around data, the modern-day commodity. Patient data are often stored only by the treating facility, leading to data silos. Thus, data availability and interoperability are insufficient because there is no common system for sharing, and as a result, retrieving and transmitting patient data can be cumbersome and time-consuming [8]. This represents a sad reality, which can be traced back to the heterogeneity in HIS and subsystems identified by the questionnaire. Physicians need to deal with delays in data transmission and spend their time sending data via outdated technologies such as fax machines or e-mail [9]. In the meantime, state-of-the-art information technologies such as deep learning have given rise to large language models, for example, ChatGPT, or Blockchain-based decentralized data storage, which are rapidly approaching a phase of usability in healthcare settings [10, 11]. Thus, technological feasibility and reality are becoming increasingly divergent.

One of the key drivers for adopting modern HIS in Germany is the need for seamless data exchange and interoperability among different healthcare providers and systems. The German healthcare landscape comprises a network of hospitals, clinics, general practitioners, specialists, and other healthcare entities. To ensure continuity of care and facilitate information exchange, standardized data formats and communication protocols are required. By applying such standards patient information, test results, medical images, and other relevant data can be shared across different healthcare settings. Associated potential cost savings will be important for increasing the efficiency of healthcare systems [8]. Considering the aging population and the soon to be aging baby boomer generation, which will lead to an overall increase in demand for healthcare services, reducing costs constitutes an unavoidable challenge [12].

The chronic underfunding of German hospitals has created an investment holdup in billions of euros for the past three decades, turning an investment gap into a technology gap [13]. However, the Hospital Future Fund (KHZG), which provides four billion euros to support digitization in hospitals, represents one ray of hope here. In addition, the recent release of the “digitization strategy for the healthcare and nursing sector” of the German Federal Ministry of Health outlines the vision for a unified digital health ecosystem by calling out strategic areas of action for the digital transformation process in the medical care sector [14]. One of the strategy´s core projects is focused on advancing the data infrastructure and, in particular, instating media-independent processes that are simplified and accelerated through automation and access to relevant data. Digital networking goes hand in hand with increasing institutional networking of the various players in healthcare and nursing, so that interoperable collaboration can emerge across care areas and professions. In concrete terms, for example, research is being conducted on implementing a secure European data space in the Gaia-X project [15]. This work focuses on the patients and direct benefits for them in individual projects, with an emphasis on data sovereignty. The respective caregivers are integrated into the ecosystem accordingly [16].

## Conclusion

HIS play a critical role in modern healthcare by facilitating the management and delivery of patient care services. In this study, we evaluated the current landscape of HIS in gynecology and obstetrics in Germany.

The results display the variety of HIS systems in use, with an average age of 9 years. The median number of different software systems used in daily routine amounts to 4, with about 41% requiring manual data transfer mainly between these systems. Still, about 17% operate in a mainly paper-based system at their hospital. Although 36% of participants perceive themselves as innovators for digital medicine and 78.4% agreed that digital medicine can help to reduce the increasing clinical workload, only 34% would recommend their current HIS to a colleague or friend.

Thus, HIS usage in the specialty of gynecology and obstetrics in Germany is characterized by heterogeneity in applied systems, outdated and slow technological progress, and lacking interoperability. Subsequently, a considerable degree of manual labor is required of healthcare professionals, resulting in a high degree of dissatisfaction with current HIS. New HIS would be desired, mainly to provide centralized documentation of medical data, facilitate compliance with legal requirements, and increase effectiveness.

In the near future, superordinate data structures will not have completely replaced previous HIS systems, but we need to pave the way for standardizing and implementing individual solutions. As a professional society, our goal must be to play a significant role in shaping the change to digitalization.

## Abbreviations

CI: Confidence interval
IT: Information technology
SD: Standard deviation
